# Real-world evidence and the future of personalized medicine: a global perspective on data, ethics, and equity

**DOI:** 10.3389/frhs.2025.1682159

**Published:** 2025-12-08

**Authors:** Alexandros Sagkriotis

**Affiliations:** Independent Consultant in Real-World Evidence and Health Data Science, London, United Kingdom

**Keywords:** real-world evidence, personalized medicine, oncology, ophthalmology, dermatology, artificial intelligence, digital health, health policy

## Abstract

Real-World Evidence (RWE) is a critical enabler of personalized medicine (PM), offering granular insights into how interventions perform across diverse, real-life populations. This manuscript, grounded in over 30 years of health data science and regulatory experience, explores the evolving role of RWE in transforming healthcare delivery—from regulatory frameworks and policy alignment to artificial intelligence (AI)-enabled patient stratification. Through real-world case examples in oncology, ophthalmology, and dermatology, the article illustrates how digital tools and data integration can enhance patient-centred care. Each vignette concludes with an “adoption path” outlining data requirements, minimal IT changes, training, and payer-relevant endpoints. The discussion critically examines risks—such as bias, opacity in algorithms, and lack of harmonization—and translates them into a pre-deployment audit checklist and an equity checklist for subgroup performance and representativeness audits. To guide global regulatory practice, a “regulatory pragmatics” checklist is proposed, covering data quality, traceability, validation, transparency, and patient voice, including patient-generated health data (PGHD). Building on the Healthcare 5.0 vision, the manuscript aligns RWE with human-centric, sustainable, and resilient pillars, highlighting IoT wearables, environmental sensors, and continuous lifestyle data streams. Policy and implementation recommendations, together with a global convergence roadmap, position RWE as a strategic tool for regulators, payers, and clinicians. The paper concludes with a call for systemic accountability: industry must innovate responsibly, regulators must approve with foresight, payers must assess tools beyond medications, and health systems must bridge infrastructure gaps. Over the next 12–24 months, measurable commitments are required across all stakeholders to ensure that PM becomes everyday care. PM must serve patients—not just science, policy, or business—and that demands leadership grounded in scientific integrity and human empathy.

## Introduction

**“**Artificial intelligence (AI) still struggles to distinguish between a man and a woman.”

“Personalised care means identifying biomarkers—especially in oncology.”

“I know I want something, but I’m not yet sure what that is.”

These are not lines from a dystopian novel or fragments of misunderstood trial protocols. They are real statements—echoed in hushed conversations and boldly shared in corporate corridors by those who present themselves as “inspirational leaders”. These remarks rarely appear in Townhall minutes or polished LinkedIn posts—but they persist. And so, I ask: as the patient of tomorrow, can I trust my health and future to those who confuse science with strategy, suppress dissent, and speed toward innovation like untrained drivers—while deftly navigating their own career paths?

Looking ahead, what awaits me as an elderly patient in my 80s diagnosed with neovascular age-related macular degeneration (nAMD)? How do I preserve my vision while managing comorbidities, aging, limited mobility, and no caregiver? As a cancer patient facing treatment-related toxicity, how do I avoid hospitalisation or treatment interruption? And if I live in a rural area with limited specialist access, how can I receive timely and accurate diagnosis of a skin condition when local expertise is scarce and waiting lists are long?

These are not hypothetical scenarios—they are common realities that transcend disease areas. The question is no longer how advanced our research & development (R&D) pipelines are, how fast we develop products, or how compelling our health economic models appear. The real question is: are these solutions truly made for me—not just for the average patient? Will they reach me wherever I live? And if costs are involved, who will share that burden to ensure not just longer life, but better life?

This mix of frustration, lived experience, and future concern drives me to write—not with resentment, but with hope. It is a call for a more accountable, compassionate, and patient-centered healthcare ecosystem. I write through the lens of a future patient—someone who has spent decades working for individuals with cancer, retinal, and immune-mediated diseases, and now expects healthcare to return that commitment by delivering smart, personalised, and human care. Care that goes beyond buzzwords and tick-boxes; care that sees patients as whole people; care that will one day knock on our own doors.

Personalised care does not exist in a vacuum. It must be grounded in the ecosystem that supports it—including the systems we use to generate and apply knowledge. That is where real-world evidence (RWE) becomes essential. Not as a vague concept, but as a practical, evolving tool to drive systemic change.

In this manuscript, we use personalized medicine (PM) as an umbrella term that encompasses both biomarker- and genomics-driven precision medicine as well as broader approaches such as person-centered care (“the right care, for the right person, at the right time”). Where regulatory agencies use alternate terminology (e.g., “precision medicine” in Japan or “personalised care” in the UK), we harmonize these under the broader umbrella while noting contextual differences. This ensures consistency across the text and Table 1.

**Table 1:** Global regulatory frameworks and strategies integrating real-world evidence (RWE) into personalized medicine (PM).

Region/Country	Authority/Body	Definition or Focus of Personalized Medicine	Use of RWE in Personalized Medicine	Key Initiatives/References	Maturity Level
United States	FDA	Tailoring medical treatment to genetic, biomarker, phenotypic, and lifestyle data	Regulatory decision-making (e.g., label expansions, post-marketing surveillance)	21st Century Cures Act, FDA RWE Framework, Oncology Center of Excellence (1, 2)	High
Canada	CADTH	Typology-based: Molecular, digital, and clinical personalization	HTA in rare diseases, biomarker-driven care, co-dependent technologies	CADTH HTA guidelines, PM Typology Briefing (3)	Moderate
European Union	EMA/EC	Designing right therapy based on genotypic/phenotypic profiles, lifestyle, environment	Adaptive pathways, pharmacovigilance, benefit-risk assessment	ICPerMed, Regions4PerMed, EMA guidance on RWE (4–7)	High
United Kingdom	NHS/NICE	Right care for the right person at the right time; person-centered and genomics-informed	Medicines optimisation, service design, shared decision-making	NHS Genomics Strategy, NICE NG5 (8, 9)	High
Australia	MSAC	HTA of co-dependent technologies and diagnostics	Early economic modeling with RWE	MSAC evaluation reports (10)	Moderate
China	NMPA/NHC	National PM strategies linked to AI and digital health	Pilot real-world data zones, oncology-focused registries	Healthy China 2030, Hainan RWD pilots (11)	Emerging
Japan	PMDA	Precision-focused early access using biomarkers	RWE in rare disease approvals, Sakigake and Conditional Approvals	Sakigake designation, PMDA real-world datasets (12)	Moderate
South Korea	MFDS	Bioethics-compliant genetic data use in oncology and rare diseases	Draft RWE guidance, genomic screening initiatives	Korean Genome & Epidemiology Study, MFDS draft guidelines (13)	Emerging

(1) In this manuscript, we use **personalized medicine (PM)** as the umbrella term, which encompasses approaches also referred to as “precision medicine”, “stratified medicine”, or “the right care at the right time”. Precision medicine is treated as a subset of PM with a narrower focus on biomarker- or genomics-driven stratification. Regional differences in terminology are preserved in the table to reflect regulator and HTA agency usage (e.g., PMDA Japan emphasizes “precision medicine,” whereas the UK NHS and NICE emphasize “right care, right time”).

(2) **Maturity levels** were added to contextualize readiness across regions. United States, EU, and UK are categorized as High maturity, with established regulatory pathways and multiple RWE-based approvals. Japan, Canada, and Australia are classified as Moderate, reflecting structured HTA or accelerated access initiatives. China and South Korea are marked as Emerging, where pilots and draft frameworks are ongoing. The absence of Africa and Latin America in this table highlights important gaps and underscores the urgency of extending RWE adoption globally.

FDA, U.S. food and drug administration; CADTH, Canadian agency for drugs and technologies in health; EMA, European medicines agency; EC, European commission; NHS, national health service; NICE, national institute for health and care excellence; MSAC, medical services advisory committee; NMPA, national medical products administration; NHC, national health commission; PMDA, pharmaceuticals and medical devices agency; MFDS, ministry of food and drug safety; HTA, health technology assessment; RWE, real-world evidence; RWD, real-world data; PM, personalized medicine.

This table summarizes the evolving definitions, regulatory uses, and national initiatives related to PM and the integration of RWE across major global regions. It highlights the diverse—but increasingly convergent—approaches used by regulatory authorities and HTA bodies to incorporate patient-centric, real-world data into policy, approval, and access pathways.

Table 1 is intended as a **decision aid** for policymakers and regulators; see **Global Convergence and Harmonization** and **Policy and Implementation Recommendations** for next-step actions.

Why RWE? Is it just another managerial trend of the past decade? A fashionable acronym on curriculum vitae (CVs)? Or does it offer a meaningful route to better evidence and smarter decisions? What do regulators and payers expect from RWE? And more importantly—how do we move from theory to tangible, lasting impact?

RWE, derived from real-world data (RWD)—including electronic health records (EHRs), claims, registries, digital health apps, and social media—is now a recognised component of health technology assessments (HTAs), regulatory decisions, and pricing and reimbursement frameworks. It supplements the knowledge generated through clinical trials and brings us closer to how medicine works in daily practice. RWE helps uncover where refinement is needed, where patient voices are missing, and how we can shift from clinical efficacy to real-world effectiveness.

This paper is both a scientific reflection and a personal testament. After three decades at the crossroads of biostatistics, real-world evidence, and drug development, I have seen remarkable progress—but also persistent blind spots. Despite rapid innovation in data science, AI, and personalised medicine, we still resist addressing the inertia and fragmented responsibility that hinder meaningful, patient-focused transformation.

If change is to be real, it must flow in both directions. Bottom-up: from all of us—researchers, innovators, patients—who embrace a mindset that looks beyond annual targets and dares to challenge convention. Top-down: from regulators, payers, and healthcare professionals who face patients at their most vulnerable and must reconcile care needs with resource constraints.

This section introduces the pressing need to rethink healthcare from the lens of the future patient. Drawing from real-life frustrations and insights built over decades of biostatistical and regulatory experience, it critiques superficial interpretations of personalised care and challenges the status quo of fragmented healthcare innovation. It positions RWE not as an administrative tool but as a necessary force to bring equity, depth, and true patient-centredness into the era of PM.

## Global regulatory perspectives on RWE in PM

As PM evolves from concept to reality, global regulatory agencies increasingly recognize the importance of RWE in supporting its clinical, regulatory, and reimbursement applications. Although definitions and frameworks vary, a unifying principle emerges: **personalized care cannot advance without understanding how interventions perform in real-world, diverse populations.**

This section surveys how major global health systems and regulatory bodies—from the Food and Drug Administration (FDA) and European Medicines Agency (EMA) to the Ministry of Food and Drug Safety (MFDS) and Canadian Agency for Drugs and Technologies in Health (CADTH)—are integrating RWE to support the development, assessment, and implementation of PM. It highlights key regional strategies, pilot frameworks, and data infrastructure investments, demonstrating a global movement toward convergence, but also noting disparities in readiness and execution. These evolving frameworks position RWE as both a policy and scientific driver of personalised care.

### United States—the FDA's precision approach

FDA defines PM as tailoring medical treatment to individual characteristics, including genetic, biomarker, phenotypic, and psychosocial data (1). Through the 21st Century Cures Act and its RWE Framework, FDA has actively incorporated RWE into regulatory decisions, particularly in oncology, cardiology, and rare diseases. FDA's 2021 progress report highlights the value of EHRs, disease registries, and wearable data in real-world regulatory applications (2).

### Canada—CADTH's typology-based evaluation

CADTH uses a structured typology to guide PM evaluation across molecular, digital, and clinical dimensions (3). RWE is particularly valued in CADTH's HTA when randomized controlled trial (RCT) data are insufficient or not reflective of real-life subpopulations. Co-development of therapies and companion diagnostics is actively encouraged.

### European union—EMA and European commission alignment

EMA describes PM as using a patient's phenotypic and genotypic characteristics to design the right therapeutic strategy (4). EMA encourages RWE use in adaptive licensing, post-marketing safety, and biomarker validation. Simultaneously, the European Commission supports PM as a systemic transformation, enabled by big data, AI, and RWD integration (5). Initiatives such as ICPerMed and Regions4PerMed highlight regional innovation and infrastructure as essential components of RWE maturity (6). A 2021 joint EMA–PCWP/HCPWP workshop reinforced the importance of multi-stakeholder alignment for effective implementation (7).

### United Kingdom—NHS and NICE

National Health System (NHS) England defines personalized care as “the right care for the right person at the right time”, incorporating genomics, digital health, and shared decision-making (8). The National Institute for Health and Care Excellence (NICE), through NG5 guidance, operationalizes this via medicines optimisation, emphasizing a person-centred approach, clinical appropriateness, and health system sustainability (9).

### Australia—RWE in co-dependent technologies

Australia's Medical Services Advisory Committee (MSAC) evaluates co-dependent technologies, such as diagnostics that enable PM therapies, using both clinical trial and RWD (10). This pragmatic HTA approach supports early reimbursement decisions and aligns closely with innovation pathways.

### China—policy-led expansion with local pilot zones

China's national strategy embeds PM within the Healthy China 2030 and Precision Medicine Initiative (2016–2030) frameworks. While lacking a unified RWE regulatory framework, cities like Hainan and Beijing have launched RWD pilot zones to support precision oncology and pharmacovigilance (11). However, challenges persist around data standardization, interoperability, and regional capacity disparities.

### Japan—accelerated innovation through Sakigake

Japan's Pharmaceuticals and Medical Devices Agency (PMDA) supports PM through accelerated pathways like Sakigake and Conditional Early Approval. RWE derived from registries and EHRs is increasingly used to complement biomarker-based approvals, especially in rare and intractable diseases (12).

### South Korea—ethical integration and infrastructure building

South Korea continues to develop a structured approach to PM by balancing innovation with ethical oversight. Recent revisions to the Bioethics and Safety Act aim to support genetic research while safeguarding patient rights (13). MFDS has proposed draft RWE guidelines for use in regulatory submissions, with early focus on oncology and rare diseases.

National initiatives such as the Korean Genome and Epidemiology Study provide a foundation for patient stratification and data-driven screening. In parallel, the Korean Precision Medicine Initiative promotes digital infrastructure, EHR harmonization, and integration of hospital registries, enabling the scalable use of RWE in real-world settings. South Korea's regulatory bodies also reference EMA and FDA frameworks to promote international alignment (14). These efforts, combined with government investment and AI integration, position the country as a rising contributor to the global personalized care ecosystem.

As shown in Table 1, global agencies vary in their approaches: FDA emphasizes label expansion and pragmatic RWE, EMA fosters adaptive licensing, CADTH applies structured HTA frameworks, and Asia-Pacific regulators are building early pilots. We explicitly reference Table 1 here to ensure that readers understand its role as a comparative framework rather than a stand-alone list.

Table 1 summarizes the global regulatory frameworks, highlighting how key agencies incorporate RWE to support the development and implementation of PM. To aid interpretation, we also indicate relative maturity levels: United States, EU, and UK demonstrate high maturity with established regulatory pathways; Japan and Canada show moderate readiness through pilots and typology-based HTA; while China and South Korea remain emerging with early draft guidance and regional pilot zones. The absence of Africa and Latin America highlights important gaps that require urgent research and policy development.

## The convergence of RWE and personalized medicine

PM promises to tailor medical interventions to an individual's genetic, phenotypic, behavioural, and environmental profile. However, clinical trials—while essential—are limited in their ability to fully capture the variability of real-life patients. RWE, derived from RWD, bridges this gap by providing population-wide, longitudinal insights into safety, efficacy, adherence, and quality of life (QoL) across diverse healthcare settings.

### Oncology: AI, digital tools, and patient reported outcomes (PRO) integration

In oncology, the integration of digital health platforms and molecular diagnostics has revolutionized the personalization of care. A notable application of digital health tools is the use of electronic PROs (ePROs) to monitor symptoms and QoL in patients with advanced melanoma. These tools enable earlier clinical intervention, support improved adherence to therapy, and may contribute to reduced hospitalizations (15). Importantly, these benefits have been demonstrated in pragmatic trials with external validation, although subgroup analyses (e.g., older patients or those with comorbidities) remain limited, highlighting the need for equity-focused validation (15).

Further, machine learning (ML) models have been increasingly used to predict treatment outcomes, survival probabilities, and toxicity responses. These include causal ML models applied to oncological RWD (16), and AI-powered stratification tools that guide clinical decision-making in breast and colorectal cancer (17, 18). Most of these applications are at the “emerging” stage: models are internally validated but not always externally calibrated across diverse populations, and subgroup performance (by age, sex, ethnicity) is often underreported. This distinction between exploratory vs. established evidence is now explicitly stated to guide readers (18, 19). ML-based diagnostic systems, trained on imaging data, also assist in early cancer detection, including melanoma, by identifying histopathological patterns beyond human capacity (19, 20).

Predictive models like those developed by Parikh et al. use EHR data to estimate 6-month mortality among patients with metastatic cancer, enabling clinicians to better align treatment with patients' goals of care (21).

### RWE building blocks—oncology

- Data sources: EHRs, oncology registries, wearable data, and ePRO platforms
- Data standards/models: Observational Medical Outcomes Partnership (OMOP) Common Data Model (CDM) increasingly adopted; HL7 FHIR for interoperability
- Curation rules: Structured abstraction of lab values, tumour stage, PROs with missing data imputation
- Identification strategy: Both associational analyses (e.g., PRO correlations) and causal ML approaches
- Validation metrics: External validation in selected datasets; calibration often limited; subgroup performance gaps remain
- Deployment constraints: Integration into oncology workflows requires EHR plug-ins, staff training, and payer alignment on endpoints such as hospitalization reduction

### Adoption path—oncology

To operationalize these tools, clinics require: (1) access to interoperable oncology EHRs and registries, (2) minimal Information Technology (IT) upgrades for ePRO collection, (3) staff training in symptom monitoring workflows, and (4) payer recognition of reduced hospitalizations and improved QoL as reimbursable endpoints. This structured pathway offers decision-makers a reproducible template for implementation. This pathway is consistent with Japan's Sakigake designation and PMDA's use of RWE in accelerated oncology approvals, illustrating how regulatory maturity can enable earlier access to innovations (12).

### Ophthalmology: AI-driven monitoring and biomarker optimization

Ophthalmology offers some of the most mature examples of PM supported by RWE and digital tools. Home-based optical coherence tomography (OCT) enables remote disease monitoring for patients with nAMD, reducing clinic visits and ensuring timely anti-vascular endothelial growth factor (anti-VEGF) administration (22). These tools are among the most established in the field: several validation studies confirm reliability of home OCT in detecting disease activity, though subgroup data for older patients living alone remain sparse (22–25).

AI-driven interpretation of OCT scans has also shown high accuracy in identifying lesion activity in nAMD, matching expert graders (23). However, most studies remain internally validated; external, multi-site calibration and subgroup analyses by ethnicity and comorbidity are not yet consistently reported, underscoring the need for equity-focused validation (18, 19). In addition, fluctuations in macular fluid volume, quantified using automated imaging tools, have been associated with changes in visual acuity (VA)—providing actionable insight into therapy adjustments (24).

Recent studies have demonstrated the prognostic value of AI models that predict long-term visual outcomes based on baseline OCT biomarkers, allowing stratified care plans (25, 26). Furthermore, large language models are emerging to assist in automated interpretation and decision support for ophthalmic imaging (27).

### RWE building blocks—ophthalmology

- Data sources: OCT imaging, ophthalmology registries, electronic health records, patient-generated visual function scores
- Data standards/models: Digital Imaging and Communications in Medicine (DICOM) for imaging, OMOP CDM for structured data, Fast Healthcare Interoperability Resources (FHIR) for interoperability
- Curation rules: Automated segmentation, harmonization of imaging protocols, missing data imputation for longitudinal monitoring
- Identification strategy: Predictive and causal ML models linking biomarkers (fluid volume, OCT features) to visual acuity outcomes
- Validation metrics: High internal accuracy; limited external validation across multi-ethnic cohorts; subgroup performance not systematically reported
- Deployment constraints: Dependence on device access, internet connectivity, and clinic workflow adaptation

### Adoption path—ophthalmology

Successful implementation requires (1) provision of home OCT devices with reimbursement models, (2) integration of AI-driven interpretation into EHR platforms, (3) training clinicians to interpret automated lesion activity alerts, and (4) recognition by payers of improved visual acuity outcomes and reduced clinic burden as measurable endpoints. The EU's ICPerMed and Regions4PerMed initiatives provide policy alignment for integrating OCT biomarkers and home monitoring into personalized ophthalmology programs.

### Dermatology: AI, teledermatology, and skin classification

In dermatology, access remains a challenge, especially in underserved or rural areas. AI-powered smartphone apps and deep learning (DL) models now offer dermatologist-level accuracy in skin disease classification, including eczema, psoriasis, and melanoma (28, 29). While promising, these remain emerging applications: most are validated against limited test datasets and frequently underperform on darker skin tones due to training data imbalance. This limitation is explicitly acknowledged as part of equity and subgroup performance reporting (20, 28, 29). These tools enable patients to self-monitor lesions and receive early alerts for suspicious changes.

AI models have demonstrated high sensitivity in diagnosing melanocytic lesions, while also supporting dermatologists in triaging cases that require urgent attention (30, 31). These developments improve early detection and reduce diagnostic delays, particularly for high-risk patients or those with limited access to specialists.

Digital dermatology platforms also assist in monitoring treatment response for chronic inflammatory diseases. Patients with chronic hand eczema, for instance, can benefit from AI tools that assess lesion severity over time, facilitating personalized adjustments in topical or systemic therapies (32). However, deployment at scale is constrained by integration into dermatology workflows, reimbursement models, and the need for longitudinal validation across diverse populations.

Moreover, recent reviews emphasize the growing role of causal AI in dermatology—not only for classification but also for understanding therapeutic pathways, patient subgroups, and outcomes (33, 34).

Here, the manuscript explores how RWD technologies have moved beyond conceptual promise to practical application across oncology, ophthalmology, and dermatology. The section presents concrete examples—such as the use of ePROs in melanoma, home-based OCT in retinal disease, and AI-powered applications for dermatological diagnosis—that demonstrate how digital tools, when integrated with RWE, can personalise care delivery, improve outcomes, and democratise access. The evidence illustrates the tangible, multidisciplinary impact of this convergence.

### RWE building blocks—dermatology

- Data sources: Smartphone images, teledermatology platforms, dermatology registries, EHRs
- Data standards/models: FHIR for clinical metadata, OMOP extensions for dermatology-specific outcomes
- Curation rules: Quality filters for patient-generated images, consensus scoring for lesion severity, and harmonized annotation protocols
- Identification strategy: DL classification models and causal inference to explore treatment response pathways
- Validation metrics: High sensitivity/specificity in controlled settings; limited external validation on darker skin tones; subgroup performance gaps significant
- Deployment constraints: Reliance on patient smartphone quality, internet access, and specialist workflow acceptance

### Adoption path—dermatology

Practical adoption requires (1) payer-approved reimbursement for teledermatology consultations, (2) standardized pathways for uploading and securing patient-generated images, (3) integration of AI lesion scoring into dermatology EHR modules, and (4) systematic validation in multi-ethnic populations to ensure equity. China's Hainan RWD pilot zones demonstrate how regulatory sandboxes can accelerate adoption of teledermatology and AI validation frameworks in dermatology (11, 35).

## Expanded discussion: challenges, risks, and future directions in PM and RWE

Reading the current regulations, my understanding—as the patient of tomorrow—is that health authorities are engaging seriously in discussions about personalised care. Despite its varied definitions, PM clearly refers to tailoring diagnostics, therapies, and prevention strategies to individual characteristics and phenotypes. This alone is enough to make a patient feel seen—not just as another average case, but as a unique individual whose care maximizes safety, QoL, and therapeutic efficacy.

However, achieving optimal outcomes requires tight collaboration between industry, regulators, payers, healthcare providers, and patients themselves. Each stakeholder has a role to play in addressing the embedded challenges, limitations, and risks associated with implementation.

### Challenges and limitations

Despite the transformative potential of PM supported RWE, several persistent structural and methodological challenges must be addressed, particularly around implementation, validation, and equity. In particular, reproducibility, equity, and lifecycle monitoring must become non-negotiable elements of any RWE program. One of the key limitations is the inconsistency and variable quality of RWD. EHRs differ substantially across geographies and systems, with heterogeneity in data capture, coding, and interoperability. This lack of CDMs impairs cross-country comparison and limits the generalizability of findings (35).

Further issues arise from EHRs, registries, and PROs, which often lack harmonised data formats and complete records (36, 37). These inconsistencies impede evidence synthesis and hinder predictive algorithm deployment in clinical settings (38). Sheffield et al. note how difficult it is to replicate clinical trial outcomes using RWD due to misclassification, missing values, and limited follow-up periods (39).

On the methodological front, transparency and reproducibility of RWE studies remain uneven. While frameworks like RCT-DUPLICATE and HARPER call for trial-like alignment in design and analysis (40), practices such as model registration and prespecified protocols are still not standard across RWE research.

Bias and opacity in algorithms represent another major risk. Many AI systems are developed on datasets lacking demographic diversity, compromising performance for underrepresented populations (41, 42). For example, dermatology AI applications frequently underperform on darker skin tones due to training data imbalances (20).

The “black-box” nature of ML systems further complicates their integration into regulated clinical decision-making environments (43). Even when model explanation techniques like SHapley Additive exPlanations (SHAP) or Local Interpretable Model-Agnostic Explanations (LIME) are available (44), their use in regulatory-grade RWE is not yet consistent (45).

Regulatory inconsistency is another constraint. Agencies like the FDA, EMA, MFDS, and HTA bodies are developing RWE frameworks, but their standards for data quality, model validation, and clinical utility vary significantly (46). Similarly, payers have different thresholds for incorporating RWE into reimbursement, especially for diagnostics and co-dependent technologies (47).

Cost considerations further complicate matters. Digital tools for home monitoring and decentralised care often fall into grey zones of responsibility between payers, providers, and patients (48). High implementation costs and the lack of long-term effectiveness data make reimbursement uncertain, even when early clinical value is evident (49, 50).

Ethical concerns regarding consent, data reuse, and algorithmic decision-making are significant. Patients are rarely aware of how their data are used after collection—particularly in decentralised or digital settings or when data feeds into predictive tools (51, 52). Even anonymised datasets can carry re-identification risks. Dynamic consent and improved data custodianship are urgently needed (53).

**Figure 1:**
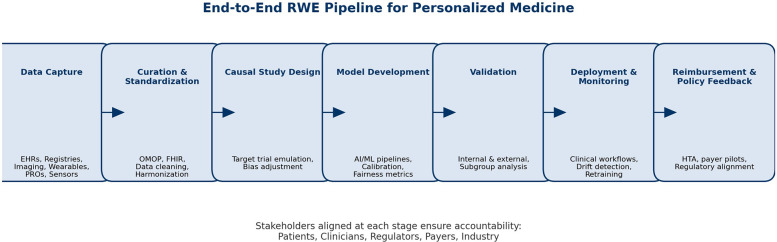
Real-world evidence (RWE) as a bridge to personalized medicine (PM). The figure illustrates how RWE can connect the traditional clinical evidence model—centered around RCTs, trial protocols, and average patient profiles—with a future of PM, AI-enhanced decision-making, and digital health. The bridge is made possible by ethical, interoperable, explainable, and patient-centric data streams such as EHRs, registries, PROs, and social media. Figure 1 should be read as a **strategic tool** for translational planning, not only an illustration.

Figure 1 illustrates a consolidated roadmap for the ethical and scalable adoption of RWE in PM, integrating strategic priorities across stakeholders.

**Figure 2:**
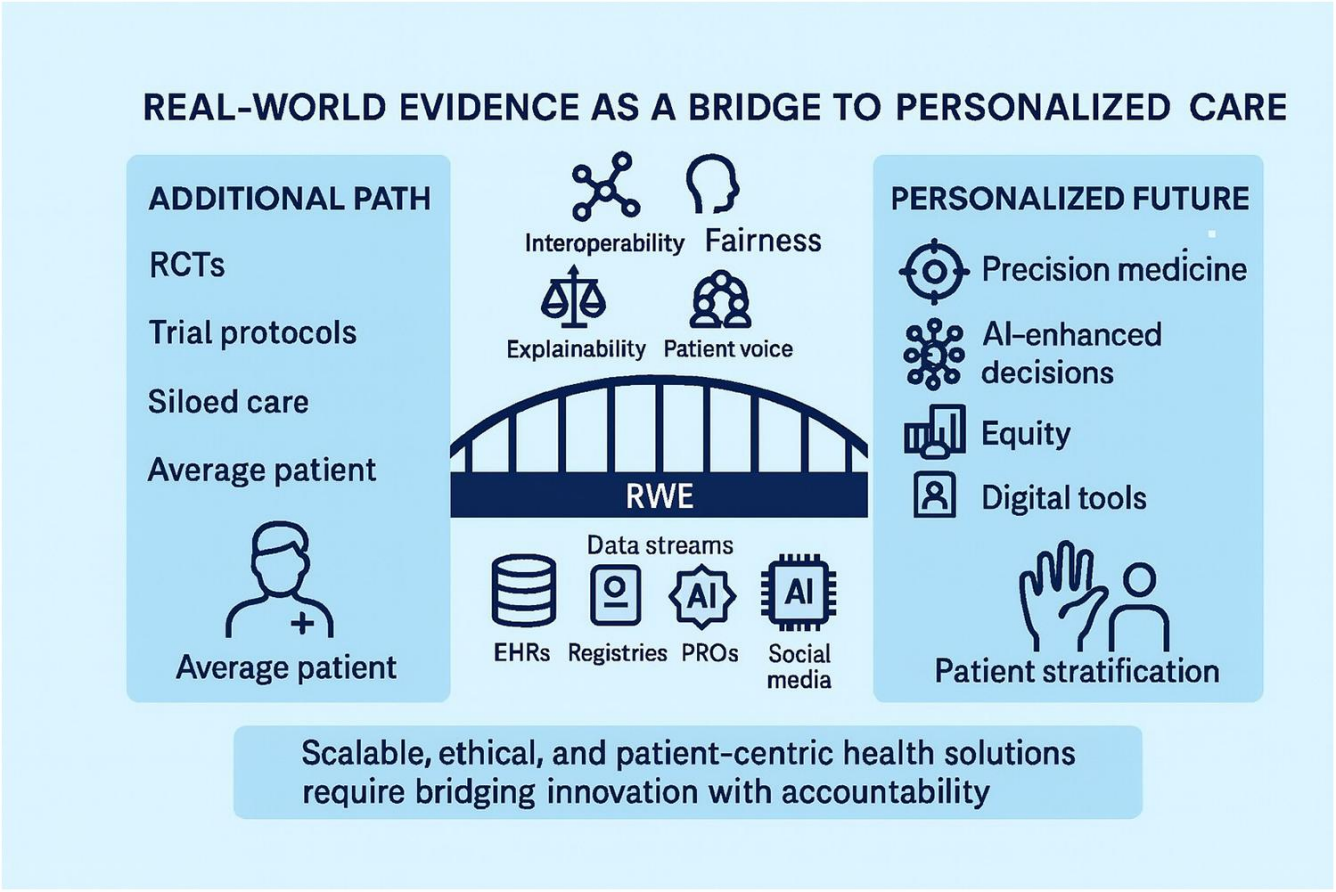
End-to-end pipeline for real-world evidence (RWE) in personalized medicine (PM). The figure illustrates the sequential stages through which real-world data are transformed into actionable evidence: data capture, curation and standardization, causal study design, AI/ML model development, validation, deployment into clinical workflows, and reimbursement/policy feedback. Each stage is aligned with stakeholder roles—patients, clinicians, regulators, payers, and industry—underscoring the shared accountability required to ensure quality, transparency, and equity. Unlike Figure 1, which emphasizes the bridge between traditional RCT-based evidence and personalized care, Figure 2 provides a more granular roadmap for operationalizing RWE in practice, making explicit the checkpoints where methodological rigor and ethical safeguards must be applied. Figure 2 functions as an **operational checklist** for teams delivering RWE in PM.

### Risk mitigation strategies

Several solutions have been proposed to mitigate the risks and limitations of RWE in personalised care.

First, aligning RWE methodologies with RCT standards is essential. Initiatives like the Real-World Evidence Transparency Initiative, use of prespecified protocols, and application of CDMs (e.g., OMOP) can increase confidence in RWE findings (54, 55).

Second, the future lies in hybrid evidence ecosystems that blend RWE with traditional trial data in iterative feedback loops for regulators and clinicians (56).

Third, regulatory-grade AI validation frameworks must be required, ensuring external validation, subgroup performance, and post-deployment monitoring (57).

Fourth, HTA frameworks should be updated to reflect process benefits like patient empowerment and improved diagnosis, in addition to clinical outcomes. Evaluations of personalised nutrition interventions suggest that multi-stakeholder dialogue and iterative modelling are essential for early-stage PM assessments (58, 59).

Finally, ethical implementation requires strong patient and public involvement (PPI). Participatory models and early patient engagement can help build trust and improve the clinical relevance of AI-enabled PM solutions (60, 61).

### Lifecycle monitoring and drift

Lifecycle monitoring of deployed models is critical. Data drift across hospitals, imaging devices, or populations can erode performance over time. We propose systematic triggers for retraining when calibration deteriorates, ensuring that predictive tools remain safe and equitable across diverse sites (18, 45, 61).

### Equity checklist

Equity in AI-enabled RWE requires consistent subgroup reporting. We propose a short checklist: (1) audit demographic representativeness of training data; (2) report subgroup performance metrics; (3) apply fairness indicators (e.g., calibration by sex, ethnicity, age); and (4) disclose limitations openly. This checklist directly addresses known performance gaps, such as dermatology AI underperforming on darker skin tones (20, 41, 45). This checklist can also serve as a procurement and payer assessment tool, ensuring that AI solutions adopted into healthcare contracts meet equity and fairness standards.

### Implementation blueprint

Implementation Blueprint for Decision-Makers: Successful adoption requires a “starter kit” that includes: (1) minimal IT integration with existing EHRs, (2) workflow redesign for ePRO and Patient-Generated Health Data (PGHD) capture, (3) staff training modules for AI-supported decision-making, and (4) payer-aligned endpoints (reduced hospitalizations, improved QoL, diagnostic accuracy). This structured pathway makes RWE operational, not aspirational (36, 54, 56).

### From clinic-centric RWE to healthcare 5.0

The evolution of RWE is moving beyond hospital and clinic walls toward a new paradigm often referred to as Healthcare 5.0. This framework emphasizes care that is human-centric, sustainable, and resilient. In alignment with Tan et al. (62), we now highlight how continuous lifestyle and environmental data must be treated as first-class signals in PM—not only genomic and clinical variables (see Table 2 for pillar-level alignment).

**Table 2:** Aligning real-world evidence (RWE) with healthcare 5.0 pillars.

Healthcare 5.0 Pillar	RWE Alignment	Example Application
Human-centric	Integration of patient-generated data (activity, sleep, mental health) into care pathways	Remote monitoring of fatigue in oncology patients
Sustainable	Continuous monitoring reduces unnecessary hospital visits and optimizes therapy scheduling	Home OCT for nAMD, reducing travel burden
Resilient	Lifecycle monitoring and retraining of AI models against data drift	Recalibration of dermatology AI apps across different skin types

RWE, real-world evidence; AI, artificial intelligence; OCT, optical coherence tomography; nAMD, neovascular age-related macular degeneration.

This table synthesizes the core reproducibility elements that enable case studies in three disease areas to be generalized beyond single studies or pilot programs. Each vignette is mapped against a set of building blocks: data sources, data standards/models (e.g., OMOP, FHIR, DICOM), curation rules, analytic identification strategy, validation metrics, and deployment constraints. By presenting these components side by side, the table demonstrates recurring patterns in how RWE is constructed and deployed. It also highlights key gaps—such as external validation, subgroup performance, and workflow integration—that remain barriers to equitable implementation. In contrast to Table 1, which provides a global regulatory panorama, Table 2 focuses on the clinical and methodological scaffolding necessary to translate digital tools and real-world data into reproducible, accountable personalized care.

Table 2 serves as a **deployment playbook** connecting Healthcare 5.0 pillars to concrete RWE building blocks.

Wearables (e.g., continuous glucose monitors, actigraphy), environmental sensors [e.g., air quality, temperature, ultraviolet (UV) exposure], and high-throughput connectivity (5G/6G) are enabling longitudinal RWE streams. These real-time data flows, when integrated with EHRs and registries under strong governance controls, allow for hyper-personalized care that adapts dynamically to patient context (62).

Table 2 provides an alignment of RWE building blocks with Healthcare 5.0 pillars, showing how data and governance expand under this new paradigm.

We propose a call to action: stakeholders should extend RWE programs to systematically capture lifestyle and environmental determinants, supported by privacy-by-design principles. This would move PM from reactive, clinic-centered care toward proactive, contextual, and equitable care ecosystems.

### Regulatory pragmatics—minimum requirements for RWE submissions

To translate narrative principles into actionable guidance, we propose a one-page regulatory checklist that can travel consistently with any RWE submission:

- Data quality: Prespecified CDM (e.g., OMOP), minimum completeness thresholds, missing data strategy.
- Traceability: End-to-end audit trail linking raw RWD to final analysis dataset.
- Validation artifacts: Documentation of internal and external validation, calibration plots, and subgroup performance metrics.
- Transparency: Prespecified protocol, public registration of study design, and clear reporting of deviations.
- Patient voice: Demonstrated incorporation of PROs or PGHD where clinically appropriate.

This pragmatic summary responds directly to regulators' demand for reproducibility while giving sponsors a concrete template for future submissions (35, 36, 63).

### Pre-deployment audit checklist for clinics and vendors

Before integrating RWE-based AI tools into practice, we recommend that clinics and vendors apply a short audit covering four domains:

- Dataset representativeness: Evidence that training data cover relevant age, sex, ethnicity, and comorbidity distributions.
- Model explainability: Availability of explanations appropriate for clinicians (e.g., SHAP plots, feature importance).
- Human-factors testing: Usability testing with clinicians and patients to assess workflow fit and risk of alert fatigue.
- Patient-facing transparency: Clear disclosure of how data are used, algorithm limitations, and channels for patient feedback.

This checklist converts high-level ethical risks into operational safeguards that are implementable across diverse healthcare settings (44, 51, 61).

### Learning health systems

RWE also enables continuous learning health systems: RWD feeds into evidence generation, which in turn updates care plans and informs regulatory and payer feedback. This data → evidence → care → policy loop ensures PM evolves continuously, not episodically (54, 57).

### Comparative readiness commentary

As noted in Table 1, readiness varies: United States, EU, and UK are high maturity; Japan, Canada, and Australia are moderate; China and South Korea remain emerging. The absence of Africa and Latin America underscores urgent needs for infrastructure, governance, and policy development in these regions (6, 11–14). See **Global Convergence and Harmonization** for mechanisms to close these gaps.

### Global convergence and harmonization

While regional frameworks for real-world evidence (RWE) are advancing at different speeds, global convergence is increasingly critical to avoid fragmented standards and duplicative efforts. Collaborative initiatives between regulators provide a foundation. For example, the **FDA and EMA** have jointly explored methodological guidance and case studies on RWE acceptance, signaling a shared commitment to reproducibility and transparency in decision-making (35–37). Within Europe, the launch of **DARWIN EU** represents a major step toward creating a federated, interoperable RWD infrastructure that can serve regulators, payers, and HTA agencies across the region (35–37).

At the global level, the **ICH E6(R3) Good Clinical Practice revision** explicitly acknowledges pragmatic and real-world designs, underscoring the growing role of RWE in regulatory science. However, convergence is uneven. While the United States, EU, and UK are classified as high-maturity regions with established pathways (Table 1), **Asia-Pacific regulators often lag behind**, with pilots in Japan, China, and South Korea not yet harmonized with US/EU frameworks. Similarly, **Canada and Australia** remain at moderate levels of integration, with RWE primarily applied in rare diseases and co-dependent technology assessments.

To bridge these disparities, **mutual learning mechanisms** are needed. Template sharing of protocols, joint scientific advice, and common data model pilots could accelerate alignment, particularly in emerging regions. Equally important is the role of HTA agencies and payers, who must converge on minimum evidence standards to support equitable patient access globally. Without such harmonization, personalized medicine risks advancing only in well-resourced settings, further widening the equity gap (35–37).

This global convergence arc highlights the importance of RWE not as a local experiment but as an international priority, requiring cooperation across regulators, HTA bodies, payers, and industry to establish common, trusted standards for evidence generation. See **Policy and Implementation Recommendations** for an actionable checklist.

### Policy and implementation recommendations

To accelerate integration of RWE into personalized medicine globally, we propose a short checklist for policymakers and regulators:

- **Shared minimum dataset:** define a core, internationally shared set of RWE elements required across submissions to improve comparability.
- **Privacy rules for continuous data:** establish harmonized, privacy-by-design governance for Internet of Things (IoT) devices (such as connected wearables, home medical devices, and environmental sensors), ensuring that lifestyle and environmental data streams (sleep, activity, UV exposure, air quality) are securely managed, consented, and interoperable across health systems.
- **Interoperability standards:** promote convergent adoption of OMOP, FHIR, and DICOM adoption to enable cross-border RWE use.
- **Equity safeguards:** require subgroup performance reporting and representativeness audits for every AI-enabled RWE submission.
- **Capacity building in emerging regions:** incentivize investment and international collaboration in Africa, Latin America, and Asia-Pacific.
- **Stakeholder accountability:** link regulatory and payer decisions to measurable outcomes in patient access, quality of care, and system sustainability.

This checklist provides regulators and policymakers with a practical starting point for accelerating convergence while safeguarding equity and patient trust (54, 56, 59).

### Next steps and future directions

To transition PM from pilot programs to routine care, the focus must shift to transparency, reproducibility, and patient-centric design. Standardising protocols, publishing negative findings, and integrating digital biomarkers and remote monitoring into practice are necessary steps (63, 64). Trust in AI systems will be enhanced by explainability tools (e.g., SHAP values), external validation, and fairness metrics (65). Embedding PPI as a foundational element, rather than an afterthought, will foster public trust and ensure ethical scalability of personalised care initiatives (53).

## Conclusion

PM is no longer aspirational—it must become everyday care. But its promise hinges on collective responsibility and intentional action.

**Industry** must commit to innovation grounded in ethics, inclusivity, and external validation of digital tools and RWD applications. **Regulators** must shift from approving only drugs to also supporting rigorously validated AI models, companion diagnostics, and infrastructural digital health tools. **Payers** must broaden their perspective beyond medications, valuing early diagnosis, remote monitoring, and AI-enhanced patient-centered insights. **Healthcare systems**, particularly those serving underserved or rural communities, must invest in the digital capacity and workforce needed to harness these innovations equitably.

Above all, **patients**—who contribute not only their data but their lives, and who often bear the consequences of our innovations—deserve transparency, empathy, and trust. Responsibility cannot be offloaded to algorithms, junior staff, or vague governance frameworks. Patients have the right to receive clear, honest explanations about the predictions that inform their care, the decisions made on their behalf, and how their data is being used.

As soon as possible, each stakeholder should commit to implementing measurable actions within their scope, in a collaborative manner with other stakeholders, keeping in mind that the key value is patients and their lives:

- **Industry:** publish protocols and validation datasets for AI models; demonstrate subgroup performance; establish equity dashboards.
- **Regulators:** harmonize minimum RWE submission standards; pilot shared templates for global convergence.
- **Payers**: include remote monitoring and AI-enabled diagnostics in reimbursement pilots; define reimbursable endpoints beyond drug efficacy.
- **Clinicians and health systems:** adopt site-readiness criteria; train staff to integrate ePROs, PGHD, and AI decision-support tools into workflows.
- **Patients and advocates:** co-develop transparency statements; participate in governance boards overseeing RWE use.

This is not simply a scientific or policy challenge—it's a moral one. The persons seated behind the screen must embody leadership, not just in title but in integrity. PM isn't about trends or talent pipelines—it's about human lives. By grounding these responsibilities in concrete actions, stakeholders can ensure that PM becomes everyday care—equitable, accountable, and patient-centered. Let us act on this understanding, starting now.

## Glossary

AI: artificial intelligence
anti-VEGF: anti–vascular endothelial growth factor
CADTH: Canadian agency for drugs and technologies in health
CDM: common data model
CV: curriculum vitae
DICOM: digital imaging and communications in medicine
DL: deep learning
EC: European commission
EHRs: electronic health records
EMA: European medicines agency
ePROs: electronic patient-reported outcomes
FDA: U.S. food and drug administration
FHIR: fast healthcare interoperability resources
HTAs: health technology assessments
IT: information technology
IoT: internet of things
LIME: local interpretable model-agnostic explanations
MFDS: ministry of food and drug safety (South Korea)
ML: machine learning
MSAC: medical services advisory committee (Australia)
nAMD: neovascular age-related macular degeneration
NHC: national health commission (China)
NHS: national health service (UK)
NICE: national institute for health and care excellence (UK)
NMPA: national medical products administration (China)
OCT: optical coherence tomography
OMOP: observational medical outcomes partnership
PGHD: patient-generated health data
PM: personalized medicine
PMDA: pharmaceuticals and medical devices agency (Japan)
PPI: patient and public involvement
QoL: quality of life
PROs: patient reported outcomes
R&D: research and development
RCTs: randomized controlled trials
RWE: real-world evidence
RWD: real-world data
SHAP: SHapley additive exPlanations
UV: ultraviolet
VA: visual acuity.
